# Sex‐specific changes in the aphid DNA methylation landscape

**DOI:** 10.1111/mec.15216

**Published:** 2019-09-22

**Authors:** Thomas C. Mathers, Sam T. Mugford, Lawrence Percival‐Alwyn, Yazhou Chen, Gemy Kaithakottil, David Swarbreck, Saskia A. Hogenhout, Cock van Oosterhout

**Affiliations:** ^1^ Department of Crop Genetics John Innes Centre Norwich Research Park Norwich UK; ^2^ Earlham Institute Norwich Research Park Norwich UK; ^3^ School of Environmental Sciences University of East Anglia Norwich Research Park Norwich UK; ^4^ Present address: The John Bingham Laboratory NIAB Cambridge UK

**Keywords:** dosage compensation, epigenetic regulation, *Myzus persicae*, sex‐biased gene expression, sex chromosome

## Abstract

Aphids present an ideal system to study epigenetics as they can produce diverse, but genetically identical, morphs in response to environmental stimuli. Here, using whole genome bisulphite sequencing and transcriptome sequencing of the green peach aphid (*Myzus persicae*), we present the first detailed analysis of cytosine methylation in an aphid and investigate differences in the methylation and transcriptional landscapes of male and asexual female morphs. We found that methylation primarily occurs in a CG dinucleotide (CpG) context and that exons are highly enriched for methylated CpGs, particularly at the 3′ end of genes. Methylation is positively associated with gene expression, and methylated genes are more stably expressed than unmethylated genes. Male and asexual female morphs have distinct methylation profiles. Strikingly, these profiles are divergent between the sex chromosome and the autosomes; autosomal genes are hypomethylated in males compared to asexual females, whereas genes belonging to the sex chromosome, which is haploid in males, are hypermethylated. Overall, we found correlated changes in methylation and gene expression between males and asexual females, and this correlation was particularly strong for genes located on the sex chromosome. Our results suggest that differential methylation of sex‐biased genes plays a role in aphid sexual differentiation.

## 1 INTRODUCTION

Sexual dimorphism is widespread in the natural world, and such differences are often underpinned by genetic adaptations that reside on the sex chromosomes (Mank, 2009; Rice, 2006). In mammals and birds, these sex chromosomes tend to be diverged between the sexes, (i.e., the X and Y, or the W and Z), alleviating some of the sexual antagonistic conflicts (Pennell & Morrow, 2013). Insects show a vast diversity of sex chromosome systems which range from the classical male heterogametic XY system in Drosophila, to ZW systems in Lepidoptera (Blackmon, Ross, & Bachtrog, 2017; Kaiser & Bachtrog, 2010). In some insect clades, such as grasshoppers, crickets and cockroaches, the original Y chromosome has been completely lost. In those species, the males carry a single X, whereas females are XX (Kaiser & Bachtrog, 2010). The absence of diverged sex chromosomes poses a nontrivial evolutionary challenge; how can a single genome code for phenotypes that are so fundamentally different as those of males and females? One possible solution is that the genes are differentially expressed in the sexes (Charlesworth, 2018; Ellegren & Parsch, 2007; Papa et al., 2017; Wright et al., 2018), and various epigenetic mechanisms have been suggested that could facilitate such expression variation (Allis & Jenuwein, 2016; Grath & Parsch, 2016; Holoch & Moazed, 2015).

Cytosine methylation is an epigenetic mark found in many eukaryotic organisms (Bewick et al., 2017; Bewick, Vogel, Moore, & Schmitz, 2016; Feng et al., 2010; Zemach & Zilberman, 2010). In mammals, cytosine methylation mainly occurs in a CG dinucleotide context (CpG) (Suzuki & Bird, 2008). However, in human embryonic stem cells (Guo et al., 2014), and human and mouse oocytes (Guo et al., 2014; Okae et al., 2014), cytosines are methylated in other sequence contexts (non‐CpG). Plants also have high levels of non‐CpG methylation that is maintained by a set of specialised chromomethylase enzymes not found in other eukaryotes (Bewick et al., 2017). CpG methylation is extensively detected throughout mammalian and plant genomes; it is often associated with suppression of the expression of genes or transposable elements, although other reasons have been suggested that could explain the correlation between transcriptional activity and demethylation (Bestor, Edwards, & Boulard, 2014). In contrast to the genomes of mammals and plants, insect genomes have sparse cytosine methylation almost exclusively restricted to CpG sites in gene bodies (Zemach, McDaniel, Silva, & Zilberman, 2010). Furthermore, rather than potentially suppressing gene expression, insect CpG methylation is associated with high and stable gene expression (Glastad, Gokhale, Liebig, & Goodisman, 2016; Libbrecht, Oxley, Keller, & Kronauer, 2016; Patalano et al., 2015; Wang et al., 2013; Xiang et al., 2010).

Social Hymenoptera have been used as a model system to study the function of insect DNA methylation and its role in phenotypic plasticity (Yan et al., 2014). However, replicated experimental designs have recently shown random between‐sample variation (low repeatability) and no evidence of statistically significant differences in CpG methylation between social insect castes in unreplicated studies (Libbrecht et al., 2016). Furthermore, DNA methylation has a patchy distribution across the insect phylogeny, having been lost in many species, and appears to be dispensable for the evolution of sociality and the eusocial division of labour (Bewick et al., 2016). Besides Hymenoptera, termites (epifamily Termitoidae) have independently evolved sociality in insects, and they have also been studied to investigate patterns of DNA methylation among castes and between the sexes (Glastad et al., 2016). This study found that methylation was considerably higher in termites than in any other social insects, and that many more genes were methylated. Development of additional model systems is therefore desirable to gain a deeper understanding of the function of cytosine methylation in insects.

Aphids have a functional DNA methylation system (Bewick et al., 2016; Hunt, Brisson, Yi, & Goodisman, 2010; Walsh et al., 2010) and are an outgroup to holometabolous insects (Misof et al., 2014), which have been the main focus of research into insect DNA methylation to date. Furthermore, aphids display extraordinary phenotypic plasticity during their life cycle (Dixon, 1977), in the absence of confounding genetic variation, making them ideal for studying epigenetics (Srinivasan & Brisson, 2012). During the summer months, aphids are primarily found as alate, asexually reproducing, females. These asexual females are able to produce morphologically distinct morphs in response to environmental stimuli. This can include the induction of winged individuals in response to crowding (Müller, Williams, & Hardie, 2001), or the production of sexually reproducing forms in response to changes in temperature and day length (Blackman, 1971b). In the case of the production of sexually reproducing individuals, sex is determined by an XO chromosomal system where males are genetically identical to their mothers, apart from the random loss of one copy of the X chromosome (Wilson, Sunnucks, & Hales, 1997). Differences between aphid morphs are known to be associated with large changes in gene expression (Jaquiéry et al., 2013; Purandare, Bickel, Jaquiery, Rispe, & Brisson, 2014), but whether or not changes in cytosine methylation are also involved is unknown.

Here, we performed the first indepth, genome‐wide, analysis of aphid DNA methylation. We conducted whole‐body analysis, rather than tissue‐specific analysis, because the principal aims of our study were to assess whether (a) the X chromosome and autosomes differ in methylation, (b) the sexes differ in methylation, and (c) methylation is correlated to gene expression. However, given that the development of males is induced by changes in daylight conditions, changes in methylation and gene expression could be due to variations in temperature and light, not due to sex. Furthermore, our females (but not our males) may contain embryos at various developmental stages, which could affect methylation (Field, Lyko, Mandrioli, & Prantera, 2004). Unless the age of individuals is standardised, this is a common caveat in these experiments. Hence, we have interpreted the differences in methylation observed between the sexes with caution. The comparison between the X chromosome and autosomes, on the other hand, reflects genuine differences which are unlikely to be biased by our experimental design. We find that asexual females and males have distinct expression and methylation profiles and that changes in methylation differ between the X chromosome and autosomes. In males, the autosomes are hypomethylated relative to asexual females whilst the X chromosome is hypermethylated. Changes in gene expression and methylation between asexual females and males are correlated, and this correlation is strongest for X‐linked genes. Taken together, our findings suggest a role for DNA methylation in the regulation of aphid gene expression, and that methylation is intrinsically linked to sexual dimorphism in aphids.

## 2 MATERIALS AND METHODS

### 2.1 Aphid rearing and sample preparation

An asexual colony of *Myzus persicae* clone O derived from a single apterous asexual female (Mathers et al., 2017) was maintained on *Brassica rapa* plants in long‐day conditions (14 hr light, 22°C day time, and 20°C night time, 48% relative humidity). Male morphs were induced by transferring the colony to short‐day conditions (8 hr light, 18°C day time, and 16°C night time, 48% relative humidity) and samples collected two months after transfer. Replicate samples were harvested from the same populations, with each replicate consisting of 20 adults, with apterous asexual females collected from the long‐day population, and males from the short‐day population. Samples were immediately frozen in liquid nitrogen prior to RNA or DNA extraction. DNA (three biological replicates per morph) was extracted using the CTAB protocol (Marzachi, Veratti, & Bosco, 1998), with the addition of a proteinase K digestion step during the initial extraction. RNA (six biological replicates per morph) was extracted using the Trizol reagent according to the manufacturer's protocol (Sigma), and further purified using the RNeasy kit with on‐column DNAse treatment (Qiagen).

### 2.2 Transcriptome sequencing

RNA samples were sent for sequencing at the Earlham Institute (Norwich, UK) where 12 nonorientated libraries were constructed using the TruSeq RNA protocol v2 (Illumina #15026495 Rev.F). Total RNA (1 µg) was enriched for mRNA using oligo(dT) beads. The RNA was then fragmented and first strand cDNA synthesised. Following end repair and adapter ligation, each library was subjected to a bead‐based size selection using Beckman Coulter XP beads (Beckman Coulter Inc.) before performing PCR to enrich for fragments containing TruSeq adapter sequences. Libraries were then pooled and sequenced on the Illumina HiSeq 2000 platform (Illumina Inc.) (v3 chemistry; 2 × 100 bp), generating between 15 and 57 million paired‐end reads per sample. RNA‐seq reads have been deposited in the NCBI short read archive (SRA) under accession number PRJNA437622.

### 2.3 Gene expression analysis

Raw RNA‐seq reads for each sample were trimmed for low quality bases and adapter contamination with trim galore! version 0.4.0 using default settings for paired end reads (http://www.bioinformatics.babraham.ac.uk/projects/trim_galore/). Gene‐level expression quantification was then performed for each sample based on the *M. persicae* clone O reference genome and gene annotation (Mathers et al., 2017), using rsem version 1.2.31 (Li & Dewey, 2011) with star version 2.5.2a (Dobin et al., 2013). Average expression and the coefficient of variation was calculated per gene for asexual females and males separately using FPKM (fragments per kilobase of transcript per million) values estimated by rsem. We also identified differentially expressed (DE) genes between asexual females and males using edgeR (Robinson, McCarthy, & Smyth, 2009) based on gene‐level expected counts estimated by rsem. Only genes with greater than two counts‐per‐million in at least three samples were retained for DE analysis and we considered genes DE if they had a fold‐change (FC) ≥1.5 and *p* < .05 after adjusting for multiple testing using the Benjamini–Hochberg (BH) procedure (Benjamini & Hochberg, 1995).

### 2.4 Bisulphite sequencing

Bisulphite sequencing library construction was performed using 500 ng genomic DNA per sample with a BIOO Scientific NEXTflex Bisulfite‐Seq Kit (Bioo Scientific Corporation) according to the manufacturer's instructions with the following modifications: genomic DNA was sheared to 200–400 bp with a Covaris S2 sonicator (Covaris Inc.) using the following settings: duty cycle 10%, intensity five, 200 cycles per burst for 120 s. The power mode was frequency sweeping, temperature 5–6°C and water level 12. Libraries either received NEXTflex barcode #24 (GGTAGC) or #31 (CACGAT). All purified libraries were QC checked with the Bioanalyzer DNA HS assay and further quantified by Qubit dsDNA HS Assay Kit (Life Technologies) before pooling as pairs. Pooled libraries were further quantified by qPCR using a KAPA Library Quantification Kit – Illumina/ABI Prism (Kapa Biosystems Inc.) on a StepOnePlus Real‐Time PCR System (Life Technologies). Sequencing was performed at the Earlham Institute (Norwich, UK) on an Illumina HiSeq 2500 (Illumina Inc.) using paired‐end sequencing FPKM (v4 chemistry; 2 × 126 bp) with a 15% PhiX spike in, clustering to 650 K/mm^2^. In total, we generated between 70 and 127 million paired‐end reads per sample.

### 2.5 DNA methylation analysis

Bisulphite treated reads for each sample were trimmed for low quality bases and adapter contamination using trim galore! version 0.4.0 with default settings (http://www.bioinformatics.babraham.ac.uk/projects/trim_galore/). Read pairs where one or both reads were shorter than 75 bp after trimming were discarded. We then mapped the trimmed reads to the *M. persicae* clone O reference genome (Mathers et al., 2017) using bismark version 0.16.1 (Krueger & Andrews, 2011). Trimmed reads were also mapped to the genome of the *M. persicae* strain of the obligate aphid endosymbiont *Buchnera aphidicola* (Mathers et al., 2017) to estimate the error rate of the C to T conversion. Reads derived from PCR duplicates and that mapped to multiple locations in the genome were removed from downstream analysis. The distribution of methylation across selected scaffolds was visualised using sushi (Phanstiel, Boyle, Araya, & Snyder, 2014).

Overall levels of methylation in a CpG, CHG and CHH sequence context were estimated directly from mapped reads with bismark (Krueger & Andrews, 2011). We also characterised CpG methylation levels of features in the *M. persicae* clone O genome based on the reference annotation (Mathers et al., 2017). Average CpG methylation levels of introns, exons, 5′ UTRs, 3′ UTRs and intergenic regions were calculated with bedtools version 2.25.0 (Quinlan & Hall, 2010), pooling data from all replicates and counting overlapping methylated and unmethylated CpGs. We also calculated per‐gene methylation levels for asexual females and males independently in the same way. To assess the genome‐wide distribution of methylated CpGs, we filtered CpG sites to those covered by at least five reads in all samples and used a binomial test to identify significantly methylated sites in each sample using the C to T conversion error rate (derived from mapping to *Buchnera*) as the probability of success and corrected for multiple testing using the BH procedure (Benjamini & Hochberg, 1995), setting the FDR at 5% (BH adjusted *p* < .05).

Methylation differences between asexual females and males were assessed using a principle component analysis (PCA) and by identifying differentially methylated (DM) sites and genes. PCA was carried out with prcomp, implemented in r version 3.2.2 (R Core Team, 2017), using the methylation levels of CpG sites significantly methylated in a least one sample (binomial test, BH adjusted *p* < .05). We identified DM sites and genes using logistic regression implemented in MethylKit (Akalin et al., 2012) which accepts input directly from bismark. *p* values were adjusted to *Q*‐values using the SLIM method (Wang, Tuominen, & Tsai, 2011) to account for multiple testing. For the site‐level analysis, we discarded CpG sites covered by less than five reads and those that fell into the top 0.1% of coverage. We considered sites significantly DM if they had at least a 15% methylation difference at a 5% FDR (*Q* < 0.05). At the gene level, we discarded genes covered by <20 reads which fell into the top 1% of coverage, and called genes as DM if they had at least 10% methylation difference and at a 5% FDR (*Q* < 0.05). A less stringent percent methylation difference was used at the gene‐level as the signal of DM may be diluted over the length of the gene body. To assess the rate of false positive methylation calls caused by random variation between samples we generated a null distribution of DM calls at *Q* < 0.05. We generated nonredundant pairs of all possible combinations of samples where an asexual female sample is grouped with a male sample (*n* = 18). These pairs were then tested across a range of percentage methylation difference cutoffs to ascertain a threshold of methylation difference. This enabled us to determine whether a site or gene is DM, controlling for the false positive rate (Figure 2a,c). At our chosen minimum methylation difference cutoff of 15% we compared using nonredundant pairs of two replicates grouped by sex (*n* = 9) with using the 18 random pairs of one male and one asexual female replicate. We found significantly more DM CpG sites (Mann–Whitney *U*; *W* = 162, *p* = 3.44 × 10^–5^) and genes (Mann–Whitney *U*; *W* = 162, *p* = 3.36 × 10^–5^) when the samples are grouped by sex than when they are grouped randomly (Figure 2b,d).

### 2.6 X chromosome identification

We used our whole‐genome bisulphite sequencing data for males and asexual females to identify X‐linked scaffolds in the *M. persicae* clone O genome assembly based on the ratio of male to asexual female coverage using a procedure similar to Jaquiéry et al. (2018). BAM files generated by MethylKit were merged for each morph using picard version 2.1.1 (http://broadinstitute.github.io/picard/) to maximise the depth of coverage. We then calculated per site sequence depth with samtools v1.3 (Li et al., 2009). The average depth of the pooled asexual female and male samples was 79× and 90×, respectively. We then calculated the ratio of male median depth of coverage to asexual female median depth of coverage for all scaffolds longer than 20 Kb, normalising male coverage to that of asexual female coverage (multiplying male median coverage by 79/90). This resulted in a clear bimodal distribution with modes at ~0.75 and ~1.5 (Figure 5a). We applied a cutoff of male to asexual female normalised median coverage ratio <1 to assign scaffolds to the X chromosome and >1 to assign scaffolds to the autosomes. To validate the coverage results, we mapped known X‐linked (*n* = 4) and autosomal (*n* = 8) microsatellite loci from Sloane, Sunnucks, Wilson, and Hales (2001) and Wilson et al. (2004) to the clone O genome with blastn and retrieved coverage ratios for their respective scaffolds.

### 2.7 Testing for correlation between changes in methylation and expression

To investigate the relationship between changes in gene expression and methylation we compared expression and methylation levels of genes in males and asexual females. Using average expression (FPKM) and methylation levels, we calculated the log_2_ FC in expression (FC_Expr_) and methylation (FC_Meth_), and tested for correlation using Spearman's *ρ* (rho). We also investigated the effect of chromosomal location (X chromosome vs. autosomes) on the relationship between gene expression and methylation using a general linear model (GLM). The GLM was formulated with FC_Expr_ as the response variable, and FC_Meth_ as a covariate, crossed with chromosome (as fixed factor). This interaction term tests whether the slopes of the regression lines of the X chromosome and autosomes run parallel.

### 2.8 Annotation of methyltransferase genes

Amino acid sequences of human DNA methyltransferase genes were blasted against annotated protein sequences of *M. persicae* Clone O (Mathers et al., 2017). The top *M. persicae* clone O hit for each gene was then used to blast against the *M. persicae* protein set in an iterative fashion until no additional genes were identified. The E value were set as 1E‐10.

### 2.9 GO term enrichment analysis

GO term enrichment analysis of specific gene sets was performed with bingo (Maere, Heymans, & Kuiper, 2005) using the complete *M. persicae* clone O proteome as the reference set. Redundant terms were then removed with revigo (Supek, Bošnjak, Škunca, & Šmuc, 2011).

## 3 RESULTS

### 3.1 Extensive sex‐biased expression between asexual females and males

**Figure 1:**
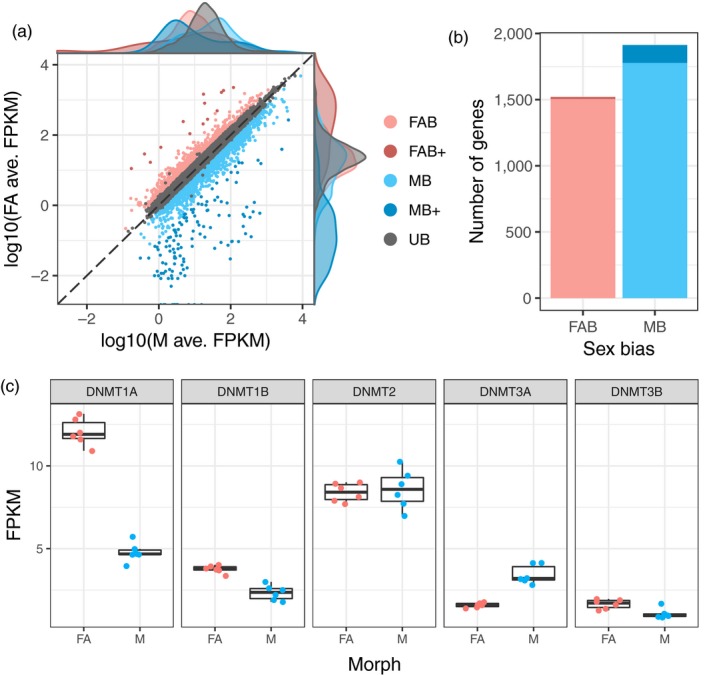
Differential gene expression between *Myzus persicae* asexual females and males. (a) Male (M; *x*‐axis) and asexual female (FA; *y*‐axis) gene expression expressed as log_10_ fragments per kilobase of transcript per million mapped reads (FPKM) averaged over six biological replicates for genes retained for differential expression (DE) analysis with edgeR (*n* = 10,334). DE genes are coloured according to the direction and magnitude of sex‐bias (see main text). UB, unbiased expression (edgeR; Benjamini‐Hochberg [BH] corrected *p* > .05 and absolute fold change [FC] > 1.5). (b) Male‐biased (MB) genes significantly outnumber asexual female‐biased (FAB) genes. (c) Asexual females and males differ significantly in expression at two out of five DNA methyltransferase genes (DNMT1a and DNMT3a; edgeR; BH corrected *p* < .05 and FC > 1.5). Given that males are derived from asexual females, we can conclude that these genes are downregulated in males. DNMT1b and DNMT3b are also significantly downregulated in males (edgeR; BH corrected *p* = 6.35 × 10^–6^ and 0.039, respectively). However, the absolute FC of these genes falls below our cutoff of absolute FC > 1.5 (FC = 1.42 and 1.35, respectively) [Colour figure can be viewed at http://wileyonlinelibrary.com]

To identify genes with sex‐biased expression in *M. persicae* clone O, we sequenced the transcriptomes of asexual females and males (six biological replicates each) using RNA‐seq (Table S1). After mapping these reads to the *M. persicae* clone O genome (Mathers et al., 2017), we conducted differential expression analysis with edgeR (Robinson et al., 2009). Genes were classified based on whether their expression was significantly biased (edgeR; Benjamini‐Hochberg [BH] corrected *p* < .05 and absolute fold change [FC] > 1.5) towards asexual females (FAB genes) or males (MB genes). We also considered the magnitude of sex bias, classifying genes as either moderately sex‐biased (1.5 ≤ FC < 10, for FAB or MB) or extremely sex‐biased (FC ≥ 10, for FAB+ or MB+). Of note is that we used whole‐body samples of multiple individuals collected at the same developmental stage, rather than tissue‐specific samples. However, given that aphids are parthenogenetic, the analysis of females may include transcripts of embryos. In contrast, the analysis of males is based on groups of single individuals, sampled at the same developmental stage. In total, 3,433 genes exhibited sex‐biased expression (Figure 1a, Table S2), representing 19% of all annotated *M. persicae* genes and 33% of all genes with detectable expression (>2 counts per million in at least three samples, *n* = 10,334). MB genes outnumbered FAB genes by 18% (1,778 vs. 1,505, binomial test; *p* = 1.02 × 10^–6^) and only a handful of FAB+ genes (15) were observed compared to 135 MB+ genes (binomial test; *p* = 1.28 × 10^–25^; Figure 1b). The relative sex‐biased expression towards males is noteworthy given that the male samples represent transcriptomes of individuals at the same developmental stage, whereas females may contain embryos of different developmental stages. The male‐biased expression is consistent with patterns of gene expression in the pea aphid (Purandare et al., 2014) and other invertebrates such as *Caenorhabditis* (Albritton et al., 2014; Thomas et al., 2012) and *Drosophila* (Zhang, Sturgill, Parisi, Kumar, & Oliver, 2007), which also show a tendency towards an excess of male‐biased genes.

### 3.2 Differentially expressed methylation genes

Next, we used our transcriptome data to investigate expression patterns of known methylation genes in *M. persicae* asexual females and males. Genome‐wide patterns of DNA methylation in animals are maintained by a toolkit of DNA methyltransferase genes (Schübeler, 2015). De novo DNA methylation is established by DNMT3 and DNA methylation patterns are maintained by DNMT1 (Law & Jacobsen, 2010). An additional homolog of DNMT1 and DNMT3, DNMT2, is responsible for tRNA methylation (Goll et al., 2006) and not involved in DNA methylation. Conservation of the DNA methylation toolkit varies across insects (Bewick et al., 2016) with DNMT1 being associated with the presence of detectable levels of DNA methylation. Aphid genomes contain a full complement of DNA methylation genes with two copies of DNMT1, a single copy of DNMT2, and two copies of DNMT3 (Mathers et al., 2017; Nicholson et al., 2015; Walsh et al., 2010). We found that DNMT1a was downregulated in males, relative to asexual females (edgeR; BH corrected *p* = 5.84 × 10^–40^, abs. FC = 2.25), and DNMT3a upregulated in males (edgeR; BH corrected *p* = 3.18 × 10^–14^, abs. FC = 2.44) (Figure 1c). DNMT1b and DNMT3b were also downregulated in males (edgeR; BH corrected *p* = 6.35 × 10^–6^ and 0.039, respectively); however, the FC of these genes fell below our 1.5‐fold threshold. In contrast, the tRNA methyltransferase DNMT2 was uniformly expressed (edgeR; BH corrected *p* = .067). These results suggest that changes in DNA methylation may be involved in the establishment of sexual dimorphism in *M. persicae*. Given that females (but not the males) contain embryos of different developmental stages, the observed difference in methylation between the sexes could also be due to a larger variation in developmental stages in the females.

### 3.3 Genome‐wide methylation patterns in *M. persicae*

DNA methylation has been poorly studied in insects outside of Holometabola and only been superficially described in Hemiptera as part of a broad scale comparative analysis (Bewick et al., 2016). We therefore first sought to characterise genome‐wide patterns of methylation in *M. persicae* before going on to investigate sex‐specific changes in DNA methylation levels between asexual female and male morphs. To characterise genome‐wide DNA methylation levels at base‐level resolution, we sequenced bisulphite‐treated DNA extracted from whole bodies of asexual females and males (three biological replicates each) derived from the same clonally reproducing population (clone O), and mapped these reads to the *M. persicae* clone O genome (Mathers et al., 2017) using bismark (Krueger & Andrews, 2011). After removal of ambiguously mapped reads and PCR duplicates, each replicate was sequenced to between 24× and 37× average read depth (Table S3), resulting in 7,836,993 CpG sites covered by at least five reads in all samples.

**Figure 2:**
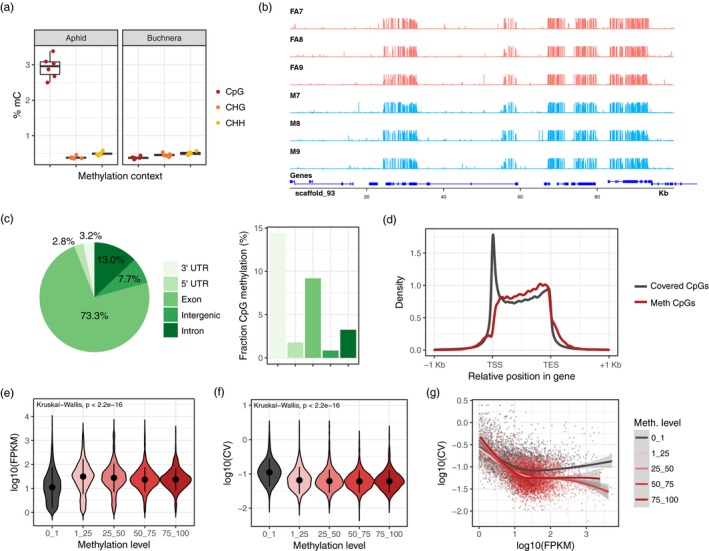
The *Myzus persicae* methylome. (a) Boxplots indicate the proportion of methylated cytosines (mC) by sequence context (CpG, CHG and CHH) for *M. persicae* and its obligate endosymbiont *Buchnera aphidicola*, which lacks a functional methylation system. (b) Example genome browser view shows the distribution of CpG methylation in asexual females and males across the first 100 Kb of scaffold_93. (c) The distribution of methylated CpGs across genomic features and the proportion of methylated CpGs in each feature. Methylated and unmethylated CpG counts were summed across all replicates. (d) The distribution of all covered CpG sites (minimum of five reads per sample) and significantly methylated CpG sites (binomial test, BH‐corrected *p* < .05) across *M. persicae* gene bodies. TSS, transcription start site; TES, transcription end site. A large spike of covered CpG sites was observed at the TSS. However, the density of methylated sites at the TSS was low contrary to what is observed in plants and humans (Eckhardt et al., 2006). (e) The distribution of RNA‐seq expression levels in asexual females (log_10_ FPKM) for unmethylated (0%–1% CpG methylation) and methylated genes (grouped in methylation bins of 25% increments). FPKM, fragments per kilobase of transcript per million. Expression values were averaged across six biological replicates and methylation levels averaged across three biological replicates. Only genes with average expression levels of at least 1 FPKM in males and asexual females were included. Dots and whiskers inside the violin plots indicate median and interquartile range respectively. (f) As for (e) but showing the distribution of variation in expression between the six asexual female RNA‐seq replicates (measured as the log_10_ transformed coefficient of variation (log_10_ CV) of FPKM) for unmethylated (0%–1% CpG methylation) and methylated genes. (g) The relationship between the mean and the CV of gene expression for unmethylated and methylated genes with a trend line for each methylation level shown as a LOESS‐smoothed line with shaded areas indicating the 95% CI. The difference between the grey (unmethylated; 0%–1% CpG methylation) and pink/red lines (methylated; >1% CpG methylation) shows that methylation is associated with reduced between‐replicate variation in gene expression, particularly in highly expressed genes. The negative correlation and downwards slope of trend lines shows that higher expressed genes are better canalized, showing less between‐individual variation in gene expression [Colour figure can be viewed at http://wileyonlinelibrary.com]

*Myzus persicae* individuals harbour an obligate endosymbiont, *B. aphidicola*. The *Buchnera* genus underwent an extensive genome reduction (Chong, Park, & Moran, 2019; Chong et al., 2019; van Ham et al., 2003), and lacks a functional DNA methylation system (van Ham et al., 2003). We made use of *Buchnera* derived reads in each sample to establish rates of false positive methylation calls caused by incomplete cytosine conversions by mapping each sample to the *M. persicae Buchnera* genome (Mathers et al., 2017) and quantifying methylation levels (Table S4). The average methylation level in *Buchnera* for Cs in any sequence context was 0.45% ± 0.68 (mean ± *SD*). This confirms that without a functioning DNA methylation pathway (van Ham et al., 2003), *B. aphidicola* cannot methylate its genes. It also indicates that bisulphite treatment of the aphid DNA was 99.55% efficient (i.e., a 0.45% false positive rate), and that it was consistent across samples. Based on this, we assessed methylation levels in *M. persicae* for Cs in a CpG, CHH and CHG context. Only Cs in a CpG context had methylation levels higher than the false positive rate in *B. aphidicola*, indicating that CpG methylation is the predominant form of DNA methylation in *M. persicae* (Figure 2a). Overall, global CpG methylation levels (2.93% ± 0.32% of Cs; mean ± *SD*) were similar to those reported in other hemipteran insects (2%–4%) and higher than in Hymenoptera (0.1%–2.2%) (Bewick et al., 2016). Exons were highly enriched for methylated CpGs relative to the rest of the genome (*χ* ^2^ = 1.07 × 10^8^, *df* = 1, *p* < 2.2 × 10^–16^), with only 7.7% of methylated CpGs occurring in intergenic regions (Figure 2b,c). Identification of significantly methylated CpG sites using a binomial test that incorporates the false positive methylation rate (derived from *Buchnera*) showed that methylation is nonrandomly distributed across *M. persicae* gene bodies. Methylated CpG sites are biased towards the 3′ end of genes despite the total number of CpG sites being much higher at the 5′ ends of genes, particularly around the transcription start site (TSS) (Figure 2d). As methylation is known to elevate the mutation rate at CpG sites (Tyekucheva et al., 2008) the difference in density of CpG sites between the TSS and the rest of the gene body suggests that methylation at the 3′ end of *M. persicae* genes has been a consistent feature over evolutionary time. This may explain the preferential loss of CpG sites at the 3′ of genes but not the TSS. Interestingly, methylation bias towards the 3′ end of genes is also seen in termites (Glastad et al., 2016), but not in holometabolous insects such as Lepidoptera (Zemach et al., 2010) and Hymenoptera (Bonasio et al., 2012; Wang et al., 2013; Zemach et al., 2010). 3′ methylation bias may therefore be a unique feature of hemimetabolous insects. In *M. persicae*, this is likely driven by high rates of methylation in 3′ UTRs (Figure 2c).

Next, we investigated the relationship between genome‐wide patterns of DNA methylation and gene expression using data for asexual females (Table S5). We found that the presence of DNA methylation was positively associated with gene expression, with methylated genes having significantly higher expression than unmethylated genes (Figure 2e). We also found that methylated genes were more stably expressed than unmethylated genes (Figure 2f), even after accounting for the higher expression of methylated genes (Figure 2g). The same patterns were also observed using male methylation and gene expression data (Figure S1). Taken together, these data suggest that DNA methylation in aphids may be involved in establishing and stabilising high gene expression, as has been suggested in corals (Liew et al., 2017) and holometabolous insects (Libbrecht et al., 2016; Patalano et al., 2015; Wang et al., 2013; Xiang et al., 2010).

### 3.4 Asexual females and males have distinct methylation profiles

**Figure 3:**
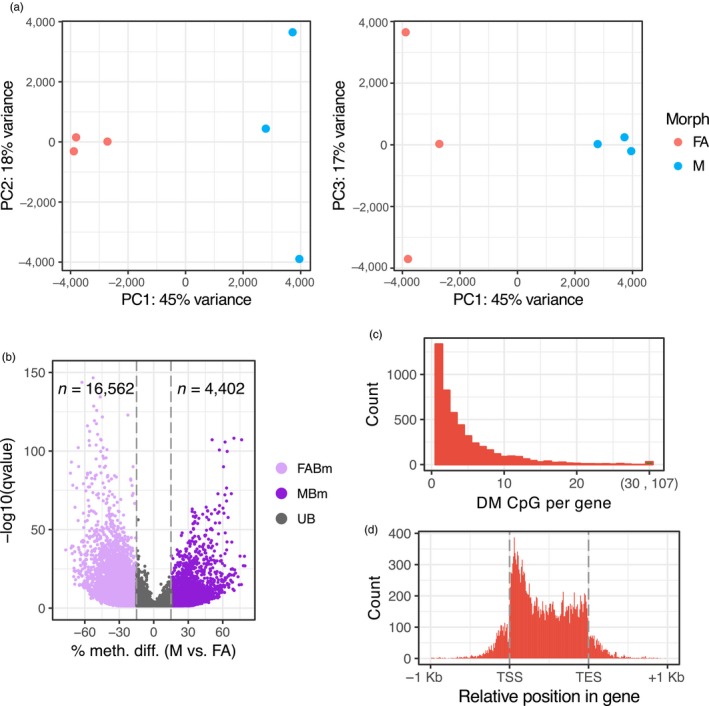
Differential methylation between *Myzus persicae* asexual female and male morphs. (a) Principle component analysis (PCA) based on methylation levels at 350,782 CpG sites significantly methylated in at least one sample. PC1 separates the samples based on sex (45% of the variation), whilst PC2 and PC3 seperate male and asexual female replicates, respectively (explaining 18% to 17% of the variation). (b) Volcano plot showing results of MethylKit (Akalin et al., 2012) site‐wise tests of differential methylation between asexual females (FA) and males (M). Methylation differences are shown for M relative to FA. Only CpG sites showing significant differential methylation (DM) (BH corrected *p* < .05) are shown. A minimum methylation difference threshold of 15% per site was applied to define a site DM between FA and M. MBm, male‐biased methylation; FABm, female‐biased methylation; UB, unbiased methylation. (c) The number of differentially methylated sites per gene (±1 Kb flanking region). DM, differentially methylated. (d) The distribution of DM CpG sites along *M. persicae* gene bodies. TSS, transcription start site; TES, transcription end site [Colour figure can be viewed at http://wileyonlinelibrary.com]

To gain an overview of methylation differences between asexual female and male *M. persicae* morphs, we conducted principle component analysis based on methylation levels of 350,782 CpG sites significantly methylated (binomial test, BH‐corrected *p* < .05) in at least one sample. Male and asexual female morphs clearly formed distinct clusters, indicating reproducible differences in global CpG methylation (Figure 3a). To further characterise methylation differences between asexual females and males we conducted site‐wise differential methylation (DM) analysis, identifying 20,964 DM CpG sites (>15% methylation difference, BH corrected *p* < .05; Table S6), 79% of which show a reduction in methylation (hypomethylation) in males relative to asexual females and 21% the opposite (Figure 3b). This was significantly higher than expected by chance (see Figure S2), and indicates that differences in methylation between asexual female and male morphs are unlikely to be due to random between‐sample variation. Rather, alterations in CpG methylation appear to be associated with the differentiation between sexual morphs in aphids. These findings are striking given the lack of evidence for significant levels of sex‐biased or caste‐biased methylation in many other insect systems (Herb et al., 2012; Libbrecht et al., 2016; Patalano et al., 2015; Wang, Werren, & Clark, 2015), although sex biased‐methylation has been observed in termites (Glastad et al., 2016).

**Figure 4:**
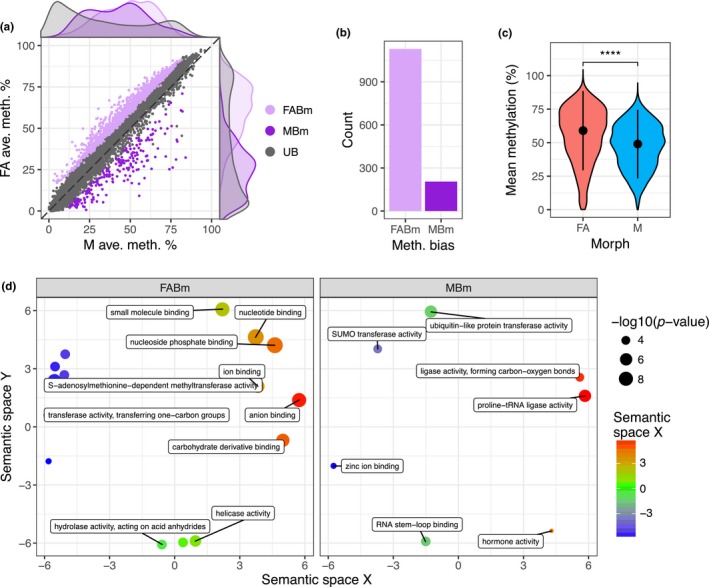
Genome‐wide changes in gene body methylation between asexual female and male morphs. (a) Male (M; *x*‐axis) and asexual female (FA; *y*‐axis) gene‐wise methylation levels averaged over three biological replicates for genes methylated >1% in at least one of the two morphs (*n* = 6,699). Differentially methylated (DM) genes (MethylKit; >10% methylation difference, BH corrected *p* < .05) are coloured according to the direction of sex‐bias: MBm, male‐biased methylation; FABm, female‐biased methylation; UB, unbiased methylation. (b) FABm genes outnumber MBm genes. (c) *Violin plot* showing the distribution of mean methylation level in FA and M for DM genes. Dots and whiskers indicate median and interquartile range, respectively; ****Wilcoxon signed‐rank test *p* < .0001. (d) Enriched GO terms relating to molecular function plotted in semantic space for FABm genes and MBm genes (for terms relating to biological process see Figure S3). GO terms are arranged in the semantic space according to their similarity in physiological and metabolically processes, as well as their functional categories, which reflects their biological meaning. A full list of enriched GO terms for each DM class and functional category is given in Table S8) [Colour figure can be viewed at http://wileyonlinelibrary.com]

Overlap analysis revealed that the majority (92%) of DM CpG sites between asexual females and males were located in gene bodies (±1 Kb), with genes having between 1 and 107 DM CpG sites (Figure 3c). These DM CpG sites were nonrandomly distributed along gene bodies, being biased towards the 5′ end of genes (Figure 3d). As such, whilst overall methylation levels are biased towards the 3′ end of genes, sites with variable methylation are more likely to be at the 5′ end. To directly correlate gene body methylation levels with gene expression, we also performed DM analysis at the gene level (Table S7). This identified 1,344 DM genes with >10% methylation difference (BH corrected *p* < .05), of which 205 showed significant male‐biased methylation and 1,129 asexual female‐biased methylation (Figure 4a,b). Considering genes with variable methylation, males have undergone a global loss of gene body methylation relative to asexual females (Wilcoxon signed‐rank test, *p* < 2.2 × 10^–22^; Figure 4c).

Gene ontology (GO) term enrichment analysis showed that asexual female‐biased methylation and male‐biased methylation genes were both enriched for GO terms relating to core biological processes, including metabolism and regulation of gene expression (Figure 4d, Figure S3, Table S8). Protein SUMOylation is enriched among genes with male‐biased methylation. This is interesting because protein SUMOylation is essential for dosage compensation of the *Caenorhabditis elegans* sex chromosome (Pferdehirt & Meyer, 2013) and plays a key role in insect development and metamorphosis (Ureña et al., 2015). Changes in methylation appear to be associated with core processes in aphid polyphenism and sex determination. Consistent with this, we also found enrichment of hormone signalling amongst genes with male‐biased methylation, with three insulin genes hypermethylated in males (two not expressed, one has male‐specific expression). Insulin receptors determine alternative wing morphs in planthoppers (Xu et al., 2015) and have been shown to interact with the core sex determination gene TRANSFORMER‐2 (Zhuo et al., 2017).

### 3.5 The X chromosome has distinct patterns of expression and methylation

**Figure 5:**
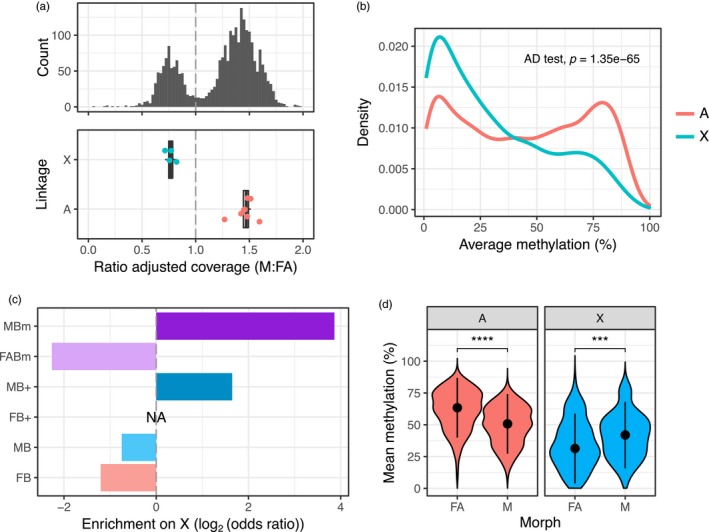
Distinct patterns of methylation and expression between the *Myzus persicae* X chromosome and autosomes. (a) X‐linked and autosomal scaffolds (≥20 Kb) in the *M. persicae* genome were identified based on the relative coverage of BS‐seq reads in males (M) compared to asexual females (FA). Given the XO sex determination system of aphids, X‐linked scaffolds are predicted to have half autosomal coverage in males. A bimodal distribution in the ratio of M to FA coverage is clearly visible (upper panel). We considered scaffolds falling in the lower coverage peak (ratio of adjusted coverage < 1) as X‐linked and scaffolds in the second, higher coverage peak (ratio of adjusted coverage > 1), as autosomal. The assignment of scaffolds to the X chromosome or autosomes was validated by comparing the M:FA ratio of coverage for scaffolds containing microsatellite markers on the X‐chromosome (blue dots) and autosome (red dots) (lower panel). (b) The distribution of gene body methylation levels for X‐linked and autosomal genes analysed in asexual females, averaged over all three replicates. (c) Observed/expected (odds ratio) counts of DM and DE genes on the X chromosome by expression or methylation bias category. The X chromosome is significantly enriched for genes with strongly male‐biased expression (MB+, ≥10‐fold upregulation in M) and genes with male‐biased methylation (MBm). (d) The distribution of mean methylation levels in asexual females (FA) and males (M) for X‐linked and autosomal DM genes (MethylKit; >10% methylation difference, BH corrected *p* < .05). Methylation levels are significantly higher in FA than M for autosomal genes, whereas M have a higher methylation than FA in X‐linked genes (d) dots and whiskers inside the violin plots indicate median and interquartile range, respectively; ***Wilcoxon signed‐rank test *p* < .001 ****p < .0001 [Colour figure can be viewed at http://wileyonlinelibrary.com]

We identified X‐linked scaffolds in the *M. persicae* genome assembly based on the ratio of male to asexual female bisulphite sequencing coverage. This approach takes advantage of the hemizygous condition of the X chromosome in males, which should result in X‐linked scaffolds having half the read depth of autosomal scaffolds (Jaquiéry et al., 2018). As expected, we observe a bimodal distribution in the ratio of male to asexual female scaffold coverage, with the lower coverage peak falling at approximately half the relative coverage of the higher coverage peak (Figure 5a, Table S9). Scaffolds in this lower coverage peak are putatively derived from the X chromosome. To validate the coverage results, we mapped known X‐linked (*n* = 4) and autosomal (*n* = 8) microsatellite loci to the clone O genome and retrieved the male to asexual female coverage ratios of their corresponding scaffolds. The coverage of these known sex‐linked scaffolds also exactly matches expectations (Figure 5a, Table S10). Using a cutoff in the ratio of adjusted male to asexual female coverage of one, we identified 748 X‐linked scaffolds and 1,852 autosomal scaffolds, totalling 68.7 and 239.7 Mb of sequence respectively (Figure S4). Scaffolds assigned to the X chromosome therefore account for 22.3% of the assembled (scaffolds ≥ 20 Kb) *M. persicae* clone O genome. This is in line with expectations given the most common *M. persicae* karyotype of 2*n* = 12 and that the X chromosome is larger than the autosomes (Blackman, 1971a). Using the chromosomal assignment of scaffolds, we were able to assign 3,110 gene models to the X chromosome and 10,768 to autosomes, leaving 4,555 (24.7%) gene models on unassigned scaffolds shorter than 20 Kb. The number of identified X‐linked genes was not different to expectations based on the assembled size of the respective chromosomal regions (binomial test, *p* = .65). However, we found that the X chromosome is depleted in coding sequence (CDS) compared to the autosomes (6.3% vs. 6.5%; *χ* ^2^ = 5,821.5, *df* = 1, *p* < 2.2 × 10^–16^). This is due to the reduced CDS length of X‐linked genes (Wilcoxon signed‐rank test, *p* = 4.2 × 10^–4^; Figure S5).

Strikingly, the X chromosome has a distinct methylation landscape compared to the autosomes (Anderson‐Darling *k*‐sample test, *p* = 1.35 × 10^–65^; Figure 5b), with fewer highly methylated genes. We also found opposing dynamics of sex‐biased methylation between the X chromosome and autosomes. The X chromosome is significantly enriched for genes with male‐biased methylation and depleted for genes with female‐biased methylation (*χ* ^2^ = 176.65, *df* = 2, *p* < 2.2 × 10^–16^; Figure 5c). Overall, X chromosome genes are hypermethylated in males (Wilcoxon signed‐rank test, *p* = 8.6 × 10^–4^; Figure 5d) compared to the genome‐wide pattern of hypomethylation (Wilcoxon signed‐rank test, *p* < 2.2 × 10^–16^). Mirroring differences in methylation between the X chromosome and the autosomes, we also found that the X chromosome was enriched for genes with extreme male‐biased expression (*χ* ^2^ = 42.38, *df* = 1, *p* = 7.5 × 10^–11^; Figure 5c), a phenomenon also observed in the pea aphid (Jaquiéry et al., 2013). Male‐biased expression of X‐linked genes is therefore conserved across two distantly related aphid species, and, at least in the case of *M. persicae*, this also extends to patterns of DNA methylation.

**Figure 6:**
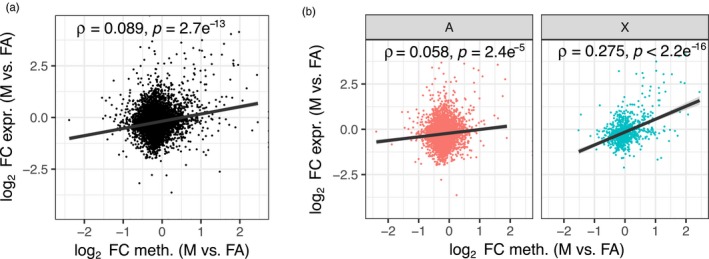
Correlated changes in expression and methylation between asexual females and males. (a) Scatter plot showing the relationship between fold‐change (FC) in gene expression and methylation between asexual females (FA) and males (M) for genes expressed (>1 FPKM) and methylated (>1%) in at least one of the sexes (*n* = 6,699). Methylation levels of genes were estimated across the whole gene body and averaged across replicates. Positive values indicate increased expression or methylation in males, relative to asexual females; negative values indicate increased expression or methylation in asexual females, relative to males. (b) The correlation between gene expression changes and methylation changes between FA and M is significantly stronger for X‐linked genes (X; *n* = 925) than autosomal genes (A; *n* = 5,272). Spearman's *ρ* was used to assess significance and strength of the relationship between change in expression and methylation for each set of genes. The trend lines indicate linear fit with shaded areas indicating 95% confidence intervals [Colour figure can be viewed at http://wileyonlinelibrary.com]

Finally, we investigated whether changes in methylation between *M. persicae* asexual females and males are associated with changes in gene expression. The relationship between gene expression and gene body methylation is an open question in invertebrates and few studies have directly tested for changes in expression and methylation. We found that DM genes were significantly enriched for DE (*χ* ^2^ = 7.84, *df* = 1, *p* = .005), suggesting that methylation changes may be involved in the regulation of at least a subset of sex‐biased genes. In support of this, we found a weak but significant positive correlation between changes in gene expression and methylation between asexual females and males when considering genes methylated (>1%) and expressed (>1 FPKM) in at least one of the sexes (*n* = 6,699; Spearman's *ρ* = 0.089, *p* = 2.7 × 10^–13^; Figure 6a). Interestingly, this correlation was driven by X‐linked genes which show a significantly stronger correlation between changes in expression and methylation than autosomal genes (GLM: *F*_1,6,185_ = 93.07, *p* < .0001; Figure 6b). Combined with recent results demonstrating a role for chromatin accessibility in the sex‐specific regulation of genes on the X chromosome and dosage compensation in the pea aphid (Richard et al., 2017), our findings suggest a key role for epigenetics in establishing patterns of X‐linked gene expression in aphids.

## 4 DISCUSSION

Here, we present the first detailed analysis of genome‐wide methylation patterns in an aphid, evaluating its importance for gene expression and sexual differentiation. We found that 3,433 genes (19% of the annotated genome) were differentially expressed between the males and asexual females, and that there was a significant excess of male‐biased genes. We also found evidence suggesting that methylation plays an important role in sexual differentiation of aphids, showing that DNMT1a and b are significantly downregulated in males, whereas DNMT3a is upregulated in males. CpG methylation is the predominant form of DNA methylation in *M. persicae* and, in contrast to other insects, exons were highly enriched for methylated CpGs at the 3′ end rather than the 5′ end of genes. Methylation is positively associated with gene expression, and in addition, methylated genes are more stably expressed than unmethylated genes. Methylation was significantly reduced in males compared to asexual females, yet remarkably, the X chromosome genes of males were hypermethylated. Given that differentially methylated genes were also significantly differentially expressed between the sexes, we propose that changes in DNA methylation are associated with *M. persicae* sexual differentiation. Our findings pave the way for future functional studies of DNA methylation in aphids, and its potential role in the remarkable evolutionary potential of these insects, and their extraordinary phenotypic plasticity.

## AUTHOR CONTRIBUTIONS

T.C.M., S.T.M., Y.C., D.S., S.H., and C.v.O. conceived the study. T.C.M. performed bioinformatics analysis with additional analysis performed by C.v.O, G.K., and Y.C. S.T.M. performed aphid morph phenotyping and extracted DNA and RNA. L.P.A. constructed bisulphite sequencing libraries. T.C.M, C.v.O., and S.H. wrote the manuscript. All authors read, edited and approved the final manuscript.

## Data Availability

Raw RNA‐seq and BS‐seq data generated for this study have been deposited in the NCBI short read archive under accession number PRJNA437622.
