# Supplementary material for: Sex‐specific changes in the aphid DNA methylation landscape
Source: Mol Ecol. 2019 Sep 22;28(18):4228–41. doi: 10.1111/mec.15216 (PMC6857007; doi:10.1111/mec.15216)
Supplement: Supplementary file 1 [file MEC-28-4228-s001.docx]

**Sex-specific changes in the aphid DNA methylation landscape**

Thomas C. Mathers^1^, Sam T. Mugford^1^, Lawrence Percival-Alwyn^2, a^, Yazhou Chen^1^, Gemy Kaithakottil^2^, David Swarbreck^2^, Saskia A. Hogenhout^1,^ * and Cock van Oosterhout^3,^ *

^1^Department of Crop Genetics, John Innes Centre, Norwich Research Park, Norwich, United Kingdom

^2^Earlham Institute, Norwich Research Park, Norwich, United Kingdom

^2^School of Environmental Sciences, University of East Anglia, Norwich, United Kingdom

^a^Current address: The John Bingham Laboratory, NIAB, Huntingdon Road, Cambridge, United Kingdom

*Corresponding authors

E-mail: c.van-oosterhout@uea.ac.uk

E-mail: saskia.hogenhout@jic.ac.uk

**Supplementary Figures**

**
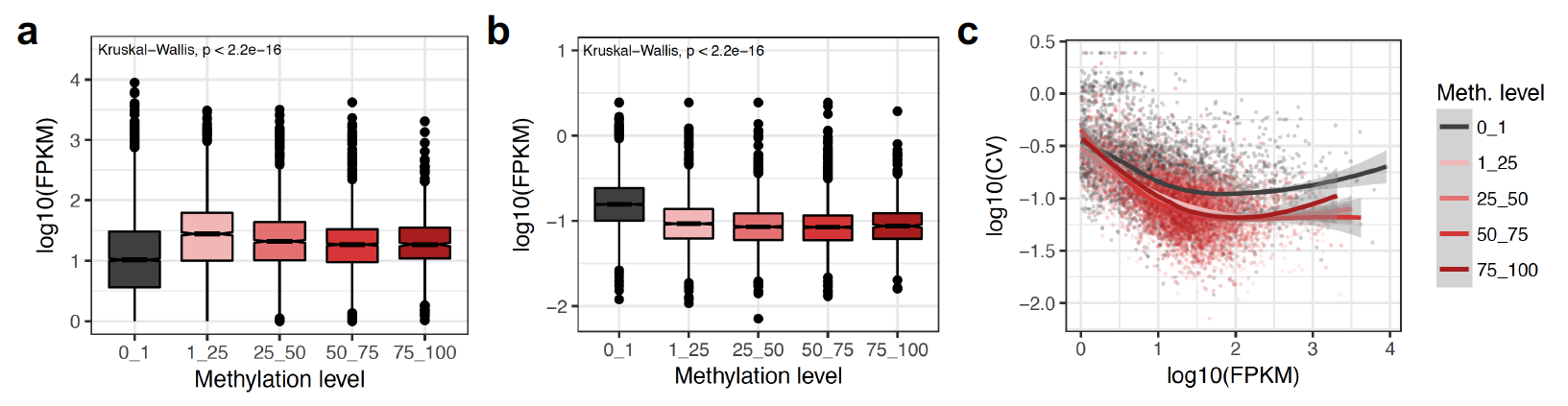
**

**Supplementary Figure 1:** (**a**) The distribution of RNAseq expression levels in males (log_10_ FPKM) for un-methylated (0-1% CpG methylation) and methylated genes (FPKM = Fragments Per Kilobase of transcript per Million). Expression values were averaged across six biological replicates and methylation levels averaged across three biological replicates. Only genes with average expression levels of at least 1 FPKM in males and asexual females were included. Dots and whiskers inside the *violin plots* indicate median and interquartile range respectively. (**b**) As for (**a**) but showing the distribution of variation in expression between the six male RNAseq replicates (measured as the log_10_ transformed coefficient of variation (log_10_ CV) of FPKM) for un-methylated (0-1% CpG methylation) and methylated genes. (**c**) The relationship between the mean and the CV of gene expression for un-methylated and methylated genes with a trend line for each methylation level shown as a LOESS-smoothed line with shaded areas indicating the 95% CI. The difference between the grey (un-methylated; 0-1% CpG methylation) and pink/red lines (methylated; >1% CpG methylation) shows that methylation reduces the between-replicate variation in gene expression, particularly in highly expressed genes. The negative correlation and downwards slope of trend lines shows that higher expressed genes are better canalized, showing less between-individual variation in gene expression.


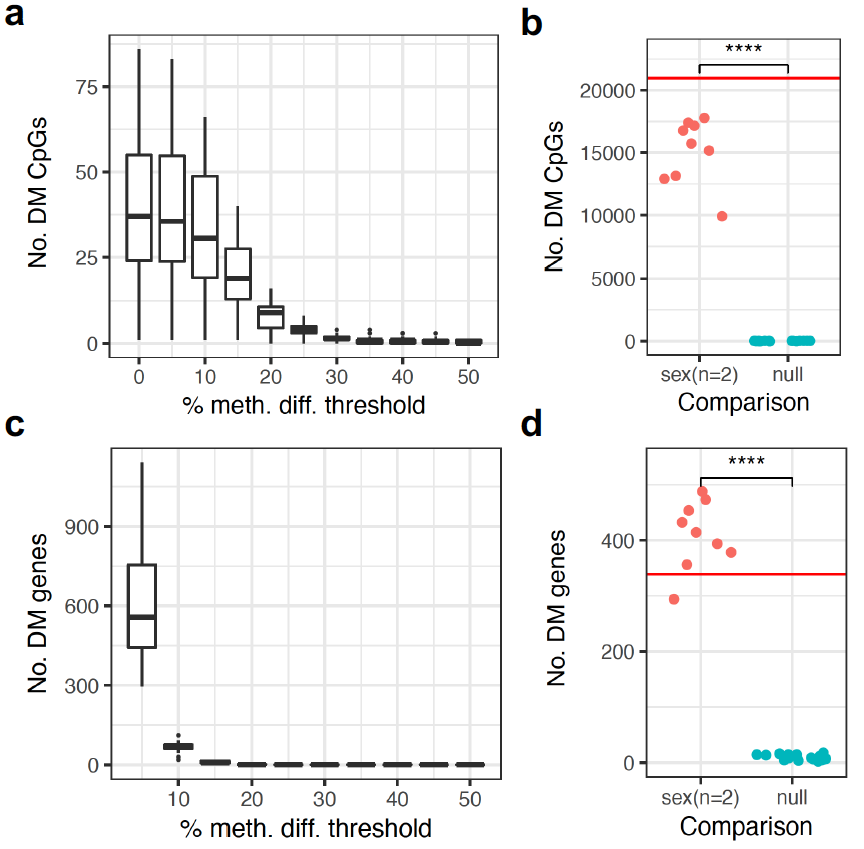


**Supplementary Figure 2:** More differentially methylated (DM) CpG sites and genes are observed between male and asexual female morphs than expected by chance. (**a**) The distribution of site-wise DM calls (MethylKit, Q < 0.05) between 18 randomised non-redundant pairs (where each group in the pair is made up of one male replicate and one asexual female replicate) at various minimum methylation difference cut-offs. At a minimum methylation difference of 15%, random differences in methylation are negligible. (**b**) At a 15% minimum methylation difference cut-off (used in the main analysis), we observe significantly more DM CpG sites when comparing all possible non-redundant pairs of two replicates grouped by sex (n=9) than when comparing between randomised non-redundant pairs (n=18) where each group in the pair is made up of 1 asexual female replicate and one male replicate (Mann-Whitney U; W = 162, *p* = 3.44 x 10^-5^). The red line shows the number of DM CpG sites when comparing all three asexual female replicates to all three male replicates at the same 15% methylation difference cut-off. (**c**,**d**) As for (**a**) and (**b**) but using gene-wise methylation levels. As for the site-level analysis, we observe significantly more DM genes when comparing pairs of two replicates grouped by sex (n=9) than when comparing between randomised non-redundant pairs (n=18) (Mann-Whitney U; W = 162, *p* = 3.36 x 10^-5^).


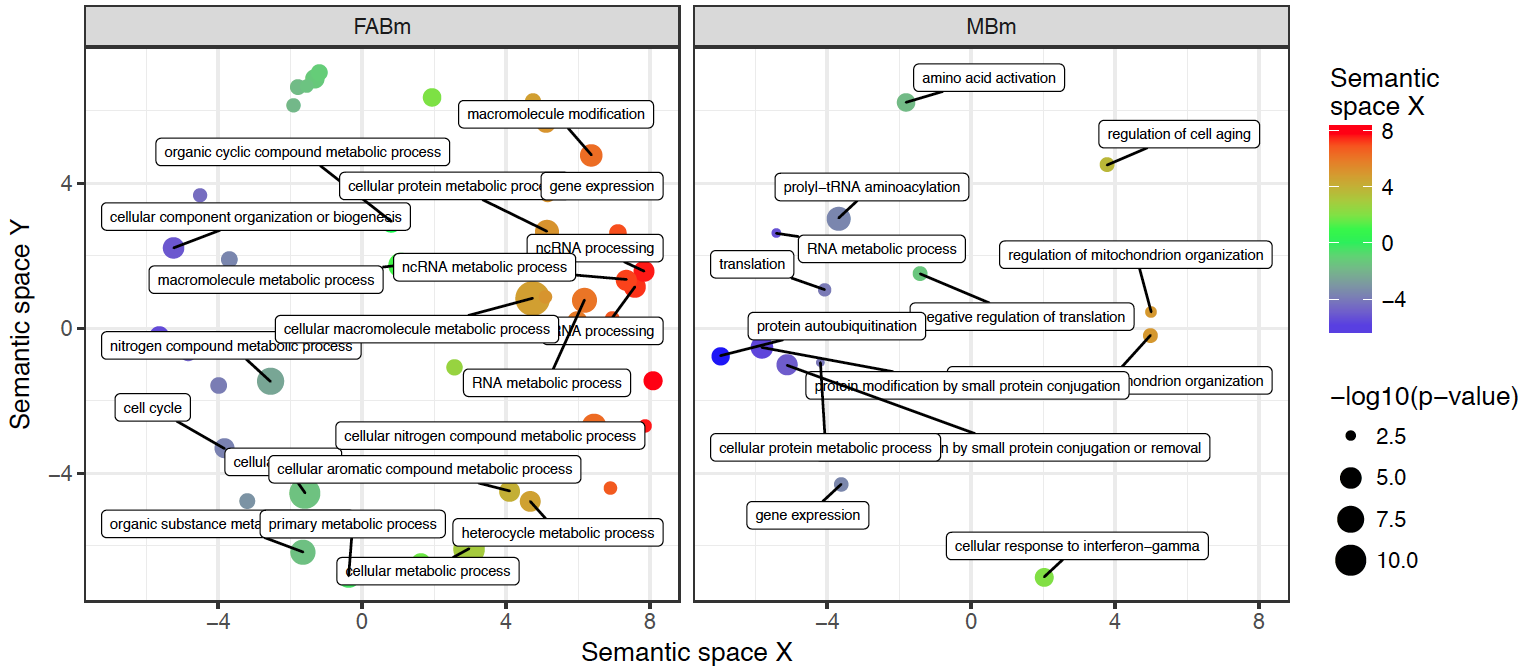


**Supplementary Figure 3:** Enriched GO terms relating to biological process plotted in semantic space for FABm genes and MBm genes. A full list of enriched GO terms for each DM class and functional category is given in **Supplementary Table 8**).

**
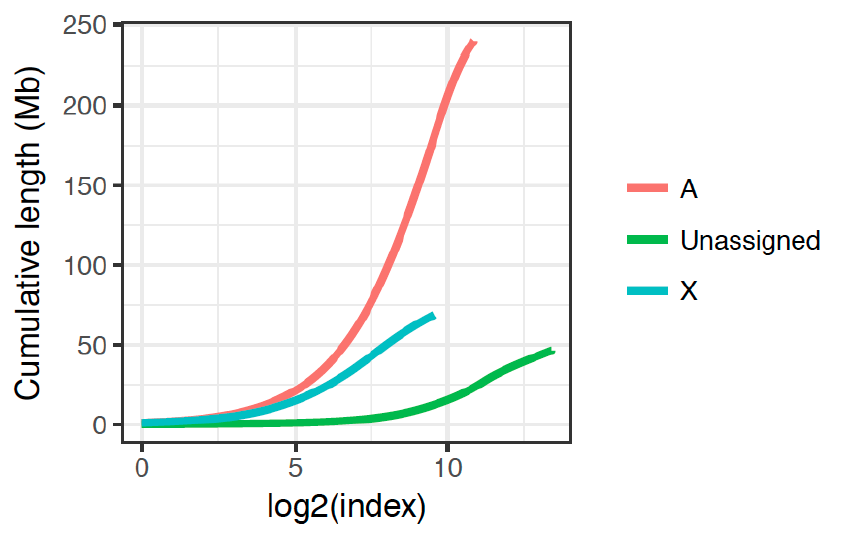
**

**Supplementary Figure 4:** Cumulative length of autosomal (239.7 Mb), X-linked (68.7 Mb) and unassigned (45.5 Mb) scaffolds.

**
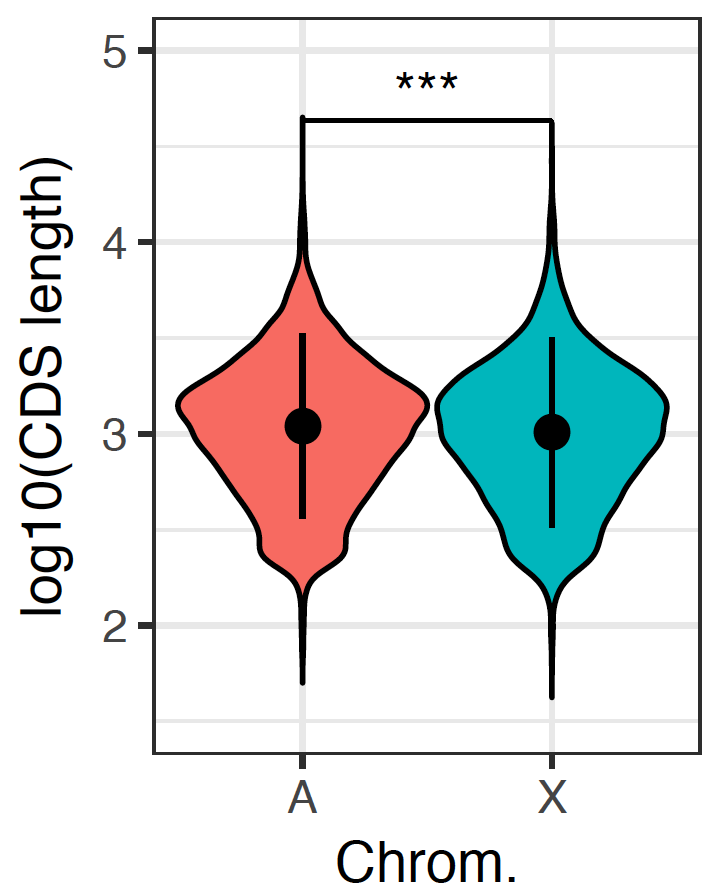
**

**Supplementary Figure 5:** *Violin plots* showing the distribution of CDS length for autosomal and X-linked genes. Where genes have alternative splice variants annotated, only the longest CDS was included. X-linked genes have shorter CDS than autosomal genes (*** = Wilcoxon signed-rank test *p* < 0.001).
